# The Hospital. Nursing Section

**Published:** 1903-08-08

**Authors:** 


### The Hospital.

### Nursing Section.

Contributions for this Section of "The Hospital" should be addressed to the Editor, "The Hospital" Nursing Section, 28 & 29 Southampton Street, Strand, London, W.C.

No. 880.—Vol. XXXIV. *SATURDAY, AUGUST* 8, 1903.

### Notes on News from the Nursing World.

### THE QUEEN AND THE NURSES FOR WEST IRELAND.

The Queen not only subscribed £50 to the Countess of Dudley's scheme for supplying nurses for the West of Ireland, but directed a letter to be written to Lady Dudley which shows the great interest she takes in the movement. Expression is given to the pleasure experienced by her Majesty in receiving the three nurses at Dublin Castle, and in hearing that they were the first appointed to join the organisation for nursing in the West of Ireland, and in conclusion it is intimated that "by sending a small donation towards the fund the Queen hopes that it may encourage others to assist in so excellent a scheme." The hope has already been fulfilled for Mr. Hugh H. Smiley, of Drumalis, Larne, in contributing the handsome sum of £5,000 says, "Since the Queen has been in Ireland she has won all hearts by her kindness and consideration for the poor and suffering, and to justify her Majesty's expressed wish I think an effort should be made to raise, without further delay, a sum sufficient for the permanent endowment of resident nurses in the poor districts of Ireland." Other donations and subscriptions have also been received by Lady Dudley, and the Duchess of Connaught has presented the beds necessary for the use of the pioneer nurses.

### QUEEN ALEXANDRA'S IMPERIAL MILITARY NURSING SERVICE.

Miss Mary L. Rannie has been appointed a matron of Queen Alexandra's Imperial Military Nursing Service, and Miss Ethel M. Denne, Miss Emily H. Hay, and Miss Dorothea M. Taylor have been appointed staff nurses provisionally. Miss Sybil R. Hughes-Hallett has resigned her appointment as staff nurse.

### THE CORONATION FUND FOR NURSES IN IRELAND.

The promoters of the movement described as "King Edward the Seventh's Coronation National Fund for Nurses in Ireland," have taken advantage of the rejoicings occasioned throughout the sister island by the Royal visit, to urge its claims upon the balances left of any funds formed for the reception of the King and Queen in various cities and towns. We were under the impression that the last had been heard of this scheme, but according to the honorary officials, the final completion has merely been held over in the hope of increasing the fund, and the Coronation Committee are sanguine that it will be in full operation by January 1st next. We have nothing to add to the criticisms which we made respecting this project when it was first adumbrated. Even if the effort to secure the augmentation of the fund by the annexation of the Royal reception balances be successful we do not think that the amount will suffice to put the scheme on a sound financial footing.

### WEST NORFOLK HOSPITAL.

The Countess of Leicester formally opened the West Norfolk and King's Lynn Hospital's new operating theatre on Thursday last week. Lady Leicester, who came in her motor-car, was met at the Globe Hotel by Miss Teppings, the Chairman's sister, who drove her to the hospital, where she was received at the front entrance by the Chairman, the medical officers and their wives, and conducted through a guard of honour, formed by the nurses, up to the new theatre. At the foot of the new staircase she was presented with a bouquet by Ethel Pitt, a patient from the children's ward, and at the top of the stairs with a silver key by the architect. When the door was opened, the Chaplain read a prayer, and a hymn was sung, after which Lady Leicester declared the theatre to be open. The building, which is quite up-to-date in every way, cost £1,500, the fittings, furniture, etc., being bought at Arnolds by the two visiting surgeons. Underneath the theatre are a spacious laundry and store-room, which were sadly needed, as there were no store-rooms. The hospital was decorated with palms, plants, and flowers, and the patients were charmed by Lady Leicester speaking to each one of them in turn.

### GARDEN PARTIES FOR NURSES.

The Lord Mayor and Lady Mayoress of Manchester entertained the nurses belonging to the hospitals and institutions in and near Manchester at a large garden party on July 28 and 29, from 3 to 7 p.m. Two consecutive days were chosen, in order that all nurses might have an opportunity of attending, and from 500 to 600 were present each day, accompanied by their friends, and the matrons, superintendents, and medical men attached to the various hospitals. The parties were held in Heaton Park, which belonged to the late Earl of Wilton, and has recently been acquired by the Manchester Corporation as a public park. On both occasions the weather was fine. The gardens and terraces round the old hall were reserved for the Lord Mayor's guests. An excellent band performed during the afternoon, while songs and recitations were also given on the terrace. Most of the nurses wore indoor uniform, and the variety of colour in the dresses, with the white aprons and caps, made up a pretty scene. Some of the largest hospitals and infirmaries sent 45 to 50 nurses each day. In addition there were the district and Queen's nurses, and also private nurses from many of the nursing homes. Refreshments of all sorts were provided, and the nurses had special electric cars to bring them to the park gates. After tea there was dancing on the lawn in front of the old hall, and an admiring ring of spectators gathered round to watch the sets of Lancers formed by the nurses of different hospitals in their distinctive uniforms. At 7 o'clock, after speeches and a vote of thanks to the Lord Mayor and Lady Mayoress, followed by much cheering and clapping, the guests made their way down the grassy slopes to the park gates, and the electric cars took them back to their hospitals, full of delight at the kind thoughtfulness which had planned such a charming entertainment for Manchester nurses.

### SUCCESSFUL BAZAAR AT GLOUCESTER.

The grand bazaar and fête on behalf of the new nurses' home in connection with the Gloucester Infirmary, the inception of which was due to the nursing staff of the institution, was considerably marred by the stormy weather of Tuesday, Wednesday, and Thursday last week, but it was none the less a great success. The commodious grounds of the infirmary were utilised for the occasion, and the numerous stalls on which an immense variety of goods were displayed were organised by the several districts of the county of Gloucestershire under the care of representatives of the best-known county families. Others gave their services in a series of entertainments and side shows. There was a formal opening each day by the Duchess of Beaufort, Lady Lucy Hicks-Beach, and the Mayoress of Gloucester respectively, to whom baskets of roses were presented by Miss Yeats, the matron, on behalf of the nursing staff. By these ladies warm tributes were paid to the nursing profession, and emphasis was laid on the necessity of providing everything necessary for their comfort and efficiency. The receipts of over £1,250 during the three days' sale exceeded the most sanguine expectations of the promoters. The cost of the new home, which is now in course of erection, together with other necessary structural work at the infirmary will be about £10,000 towards which some £6,000 had been locally subscribed prior to this special effort of the nurses.

### LIBEL ACTION BY A NURSE.

This action is reported on another page, and was settled by agreement. The defendant was the matron of the Queen Victoria Nursing Institution, Wolverhampton, which supplies the middle classes with trained nurses for members of their family when sick. There is also a nursing home connected with it, and we observe that the institution contributes over £200 a year to the District Nurses' Fund out of the profits. Seeing that it is essential to the success of the Queen Victoria Nursing Institution that it should offer the fullest safeguards to the public in regard to the character of the nurses which it supplies to private families, we are glad to hear that the committee have throughout loyally supported their matron, Miss Loveys, in the course she was called upon to take as a matter of duty, and we hope that they will take upon themselves the entire cost of defending the action. To do less than this will hardly satisfy, we imagine, public opinion in Wolverhampton, which must fully appreciate the importance of maintaining the highest standard amongst the staff of private nurses at the Queen Victoria Nursing Institution.

### DOMESTIC SERVANTS AS NURSES AT SWINDON HOSPITAL.

The report presented by the medical superintendent of the Swindon Hospital Board is melancholy reading. The nursing staff of the temporary Isolation Hospital, which was closed at the end of 1902, nominally consisted of a matron, charge nurses, assistant nurses, and probationers, but the medical superintendent states that "it was found impossible to maintain it on these lines so as to have efficient nursing." The report proceeds, "Reliable charge nurses and assistant nurses had not been found available, and even probationer nurses had been very difficult to engage, and were recruited very largely from the ranks of domestic servants." It appears, however, that in spite of the comment of the medical superintendent, that "the character of the work is not understood by the girls applying for it," the matron has still to depend upon this kind of material for the formation of the staff of probationers, who, according to the medical superintendent, are to carry on the work of nursing in future, because neither reliable charge nurses nor assistants can be obtained. This is a bad look-out for the patients at the Swindon Isolation Hospital, and there is another aspect of it which merits attention. The probationers of the domestic-service class will, we conclude, in due time be given a certificate, armed with which they will claim to be fully-qualified nurses, thus augmenting the difficulties in the way of raising the standard of nursing in the country.

### TRAINED NURSES ON STEAMERS.

Some time back articles appeared in our columns advocating the need of a trained nurse on board the large steamers of the various shipping companies, more particularly those conveying passengers to and from tropical countries. Since then we have frequently received inquiries from nurses desiring to obtain positions as a sick nurse or stewardess with hospital training on a "Liner." Hitherto the openings have been few and far between, but we understand that a movement in the direction of influencing the companies to permit a trained nurse to be carried on passenger ships is on foot, aud that it has met with considerable encouragement. We learn that it is proposed to form an association of lady nurses especially for the use of those vessels who desire to employ one, the nurse to be fully trained and certificated; to attend all passengers irrespective of class and without fee; to act in accordance with, and under the direction of, the ship's doctor; and to enter into an agreement with the steamship company for twelve months. On their side the company to agree to supply a distinctive uniform for the nurse; to provide a cabin with full board for her; and to allow her certain leave of absence during the continuance of the agreement. For each nurse supplied it is proposed that the shipping company should pay the association a small yearly sum. Of course the success of the scheme, which is originated by Miss Kate Penn, a fully trained nurse, and will be managed by a committee, depends mainly upon the attitude of the shipping companies.

### DISTRICT NURSING IN RICHMOND.

The enthusiasm manifested at the meeting held at Richmond, Surrey, for the purpose of sanctioning the affiliation of St. John's Ambulance Nursing

Guild with the Queen Victoria Jubilee Institute, promises well for the future of the organisation. A very influential general committee was formed, and also a strong executive committee on whom will largely fall the burden and heat of the day. Several of the speakers referred to the position of Kingston, which already possesses four district nurses and a home. Richmond, with a population of more than 24,000, will certainly need more than the one nurse engaged, and we agree with Mr. Harold Boulton, who addressed the meeting, that there should be no difficulty in raising money for two or three nurses, if the people give according to their means. The Board of Guardians might reasonably be asked to contribute, but the bulk of the income of the Association will have to be derived from the yearly subscriptions of all sorts of persons, not excluding the very poorest.

### THE CHIEF OFFICER OF A TRAINING SHIP INFIRMARY.

The proposal to appoint a sick-berth steward and his wife as chief officers of one of the training ship infirmaries of the Metropolitan Asylums Board, in place of the matron, who is resigning on the ground of ill-health, is distinctly of a retrograde character. It is contended by the committee of the board, which reported in favour of the change, that it would increase the efficiency of, and the discipline at, the infirmary. But we believe that with such a large number of boys it is desirable, not only for the sake of the nursing, but also on account of moral influence, that there should be a highly-trained matron in charge. The difficulties, which it is alleged, have been experienced in the past owing to the failure of a female officer to exercise discipline and control over the boys when a number of them have been sent to the infirmary, and of getting a porter to be subordinate to the matron, would, we do not doubt, absolutely disappear if the board appointed a thoroughly well-qualified lady as chief of the staff.

### HALIFAX UNION HOSPITAL.

An attempt to put back the clock was made at the last meeting of the Halifax Board of Guardians, who had under consideration the question of the reduction of the staff at the new Union Hospital. A resolution in favour of the reduction, however, only found two supporters, the remainder of the Board agreeing with the Chairman of the Hospital Committee that eight fully-qualified nurses and thirty probationers were not too many for wards containing 361 beds. It is curious that one of the reasons given for bringing forward the rejected motion was that in the new hospital there is every appliance calculated to help nurses in the discharge of their duties. This suggests a policy of taking away with the left hand what the right hand gives; whereas the adoption of modern methods in hospitals almost invariably needs to be accompanied by an increase, instead of by a diminution, of the nursing staff.

### ANOTHER INDIAN ORGANISATION.

It is stated in the daily press that a scheme of a comprehensive nature, under the auspices of Lady Curzon, is being worked out at Simla, for the provision of nurses for Europeans in India. The promoter, it is added, hoped that this scheme will have Government support, and that an organisation may be created for the supply of nurses in each province.

### THE TRAINING OF A COUNTY NURSING SUPERINTENDENT.

A point of much interest was raised at a meeting of the Northamptonshire District Nursing Association, at which the qualifications of a lady who was suggested for the post of county nursing superintendent were discussed. It was urged on her behalf that she was highly recommended by Queen Victoria's Jubilee Institute, and that she had been trained for a year at King's College Hospital, for a year at the South Western Fever Hospital, and for two years at Durham County Hospital. She has since had experience as district nurse at Penzance, Paddington, and Bolton, and also at Plaistow Maternity Home. We do not doubt that she is a thoroughly capable nurse. But we are not surprised at the contention of Lady Knightley, who argues that a county superintendent should at least be a fully-trained hospital nurse with three consecutive years' training in one hospital. Lady Knightley says that an organisation which aims at encouraging fully-trained nurses, should have a superintendent with the highest credentials, and it was wisely decided to refer the whole matter to the Executive Committee. A course of training which might justify the choice of a person for a position of minor authority, does not necessarily satisfy essential requirements in the case of a person who has to fulfil the duties of county superintendent; and it might be awkward if fully-trained hospital nurses should demur to work under a chief whose training has not been as orthodox as their own.

### DIED IN HARNESS AT ASHBURTON.

We regret to hear of the death of Mrs. Salter, the district nurse at Ashburton, on Sunday, after a few hours' illness. The deceased had faithfully discharged her duties for 11 years, paying thousands of visits annually to the patients in the town. The end came with painful suddenness. Up to Saturday night Nurse Salter had been engaged in her responsible work, and at a late hour on that day she was seized with a painful illness, to which she succumbed within about 12 hours. When the news became known much sorrow was expressed, and there were references to the sad event at various places of worship in the evening. Mrs. Salter will be missed by all classes, particularly by the poor people, with whom her genial manner and general kindness had made her very popular, while the District Nursing Association have lost a devoted servant.

### SHORT ITEMS.

The offertories at the services in Westminster Abbey on Sunday next will be given to the East London Nursing Association, and on Sunday, the 30th, to the Westminster District Nursing Association.—Under the will of the late Mr. Henry Bloom Noble, of Villa Marina, Douglas, Isle of Man, the sum of £5,000 is bequeathed for the purpose of adapting Clifton House as a nurses' home in connection with Noble's Isle of Man General Hospital and Dispensary, or building a nurses' home on the site of Clifton House, and enlarging the hospital.

### The Nursing Outlook.

"From magnanimity, all fear above;
From nobler recompense, above applause,
Which owes to man's short outlook all its charm."

### THE PRISON QUESTION.

The well-intentioned effort to supply the Prison Commissioners with a staff of warders of the trained-nurse type which we made on this page some months ago has failed; and we cannot believe that the failure was solely with the nurses. For it so happened that the article caused over two hundred nurses to write for further particulars to the Home Office, apparently to the distress of the clerks only. For not one of those applicants has been appointed as a probationer warder. Now out of 200 nurses surely some must have been fit for prison work if the conditions offered were suitable and if the Commissioners really did consider that "candidates with qualifications as nurses are regarded as desirable," as they themselves declared. The salary—commencing at £45 and rising to £120 is favourable compared with many nursing salaries. To our knowledge the applicants included many nurses who were actuated by the very highest motives; who did not consider salaries, who were ready to face any discomforts, whose one object and aim was to benefit fallen humanity, to try and help the most unhappy and most unfortunate and helpless of their sex. But man and red-tape stood in their way, and their efforts were useless. And what has man and his red-tape performed that he can thus afford to reject woman's offer to help her sister woman?

The report of the Prison Commissioners for the year 1902 has been issued since our last article was written, and from this report we learn that during last year there were 118,027 men sent to prison, of whom 61,373 had been previously convicted—52 per cent.; and that 48,232 women were sent to prison, of whom 34,080 had been previously convicted—70 per cent. Surely this is a justification of our plea that much more ought to be done of a saving and reforming character for women in prison. We know no stronger condemnation of the present system possible than this fact that out of every hundred women discharged from prison some seventy return again, and that the prison is indeed a place—as the chaplain of Bristol gaol once stated—for the manufacture of criminals. Let us consider what is done at present. All the religious and educational side of prison life is in the hands of the chaplain, and in a woman's prison the chaplain cannot visit alone—he has to be always accompanied by his schoolmistress; this is very right, but it is a continual drag on his work. If a prisoner be young and is in on a long sentence, and is not up to the level of Standard IV. in a Board School, she may have to submit to a little teaching weekly from the schoolmistress in reading, writing, and arithmetic, but otherwise she is simply put to do what work she can; she is dressed in a sloppy and hideous fashion, she is confined in an ugly and dreary cell, and she attends chapel services, and before she leaves is visited by one of the lady visitors who is connected with a discharged prisoners' association, and who will offer to help her on leaving. Suppose a woman goes in who is used to laundry, she may be put into the laundry to work; but the chief labour for the ordinary, helpless, hopeless woman prisoner is to scrub the long stone passages and wash down the stairs. The work is not apportioned with a view to teaching the woman what may be useful to her in after life, but only as so much "hard labour" to be done as part of the punishment.

Now let us look at the Woman's State Reformatory outside Boston, U.S.A., over which that splendid woman Mrs. Ellen C. Johnson presided for so many years. To begin with, the report is signed by five commissioners of whom two are women; in England all prison commissioners, inspectors and chaplains are men. The physician and chaplain at the Woman's Reformatory are both women. The report is for the year 1898 and it states that the prison population is falling, being less than the previous year by 27, and it states particularly that there are 112 less commitments for drunkenness and that that offence is steadily declining. Out of 305 women in the prison, 48 had been previously convicted, but the vast majority of previous convictions had taken place in other countries—many of them in England before the women emigrated. There are no "cells" in the Boston reformatory; there are nice bright rooms with flowers and pictures, and five evenings a week there is an "evening school" held in common so far as the "grade" goes, and there is plenty of bright, cheerful talk, and training all the same in self-control and self-denial. The superintendent reports that the women prisoners voluntarily gave up their July 4th holiday to work for the Bay State Hospital ship. In fact, the whole aim is to humanise, to help, to send forth reformed, these unfortunate women.

Quite recently when Mr. Ernest Flower put this question in the house of Commons:—

"To ask the Secretary of State for the Home Department, whether, in view of the fact that over 70 per cent. of the female prisoners in the gaols of this country have been previously convicted, he will consider the desirability of augmenting the lectures given by lady visitors last winter by practical teaching in laundry, cookery, and hygiene, which would fit the prisoners on leaving to earn their living; also whether he would again consider the advantages to be gained by the appointment of a female prison inspector,"

the reply was practically a negative, and an expression of belief that all that was necessary was being done. We know that Lady Battersea occasionally gives an excellent address on hygiene and temperance and so on to a select few of the 800 women in Holloway, but this is a mere drop in the ocean. And we notice in the arrangements for the further classification and teaching of male prisoners that it is not proposed they should be taught carpentry by an occasional lecture from Lord Battersea. But so long as all the prison commissioners and prison inspectors in England are men, our correspondent urges, "it seems useless to plead for women"; and as a matter of fact there are questions of cleanliness, chastity, etc., connected with the prisons which women can best deal with. It is a pity Mr. Ernest Flower did not carry the point of a woman inspector; it is also matter for grave regret that none of the many nurses who applied were appointed warders.

### Lectures on Ophthalmic Nursing.

By A. S. Cobbledick, M.D., B.S.Lond., Senior Clinical Assistant and late House-Surgeon and Registrar to the Royal Eye Hospital.

### LECTURE XVI.—COMMON DISEASES OF THE IRIS. IRITIS.

Inflammation of the iris and the troubles that may follow it are very common. If a correct diagnosis is made and appropriate treatment adopted, many of the sequelæ which are met with should never appear.

When the functions of the iris and its delicate mechanism are considered, you can readily understand that an acute inflammation, which, as elsewhere, is accompanied by swelling and exudation must seriously impair its action; we shall deal with the results later.

*Causation.*—This is not always easy to determine; many cases seem to be precipitated by cold, but there is probably in all these cases some constitutional fault which predisposes to an attack. The most common predisposing causes are syphilis and rheumatism; another frequent cause is traumatism. More uncommon causes are diabetes, the gonococcus diplococcus, typhoid fever, small-pox or pneumonia.

*Symptoms.*—When a case of iritis first comes under notice, the patient usually makes two complaints, viz., pain and impairment of vision in the affected eye.

The pain varies in degree, and is not always proportionate to the acuteness of the attack; it is neuralgic in character, becoming worse at night, and is referred to the brow or temple of the same side and not to the eye itself.

The pain may be so severe as to cause serious impairment of the general health through want of sleep and appetite for food. The vision becomes dim through the turbidity of the aqueous humour caused by exudation from the inflamed iris mixing with the aqueous; in some cases the cornea and lens are also at fault on account of an accompanying keratitis and through pigment being deposited on the anterior surface of the lens; in severe cases vitreous opacities are not uncommon. Photophobia is not usually present, but it may be a valuable early sign, if it can be demonstrated that the cornea is quite unaffected. Lachrymation is for the most part present, but is not as considerable as in corneal affections.

*Signs of Iritis.*—These vary somewhat according to the type of iritis present. There are three varieties of iritis, viz.:—

1. Simple plastic, characterised by the formation of an exudation which tends to bind the margin of iris down to the lens posteriorly.

2. Serous iritis in which the exudation is more fluid, is of a serous character and passes into the anterior chamber.

3. Parenchymatous iritis; in these cases the substance (parenchyma) of the iris is affected.

Signs found in all these forms of iritis are:—

*a.* More or less injection around the periphery of the cornea.

*b.* Alteration in the colour of the iris found by comparing the two irides; it must be remembered that a difference in the colour of two irides in the same individual may be a congenital peculiarity. This alteration in colour is very apparent in light irides; the affected iris becomes deeper in colour and of a greenish tinge.

In the case of brown irides there is often great difficulty in noting a change of colour unless the iritis is well established. A more safe guide is the presence of pigment deposited on the anterior capsule of the lens and an alteration in the pattern of the iris. The mobility of the iris to light stimulus is at first diminished through the swelling caused by engorgement of the blood-vessels and by some spasm of the sphincter, and later may be completely abolished by the exudation thrown out causing adherence of the iris to the anterior surface of the lens. In serous iritis there is not so much tendency to the formation of adhesions, the process is not so acute, and the course the disease runs is of longer duration than that in the plastic variety. In addition, the serous iritis is characterised by a peculiar form of keratitis, termed K. punctata from the appearance of a number of brown spots on the back of the cornea; they are always in the lower segment, and are arranged in a triangle, which has its base at the periphery of the cornea. Serous iritis is also more frequently accompanied by inflammation of the ciliary body.

*Prognosis.*—In the majority of cases an ordinary acute attack runs a course of about two or three weeks, and with appropriate treatment the prognosis may in most cases be described as favourable. Some few cases resist treatment for months. As regards tendency to recurrent attacks one cannot speak with much certainty, but a thorough course of treatment, during the first attack and afterwards, directed towards the constitutional taint is the best preventative. The effect on vision depends largely on whether the case is taken in hand early or allowed to run its course for a week before treatment is sought. The intensity of the attack must also be considered; some cases are so acute that the inflammation, in spite of treatment, spreads to the posterior three-fourths of the eyeball and leaves much impaired sight.

*Diagnosis.*—If attention is given to minutiæ, mistakes should be very infrequent. A common mistake often made by beginners, who only make a superficial examination, is to confuse conjunctivitis and iritis. In the former case, however, the vessels which are engorged are superficial and can be moved with the conjunctiva. In addition to this, the lids are always gummed together on waking in the morning if conjunctivitis is present. A more serious mistake is to confuse acute glaucoma and iritis. They have signs in common, viz.:

1. Injection of the deep vessels causing the white of the eye to appear red.

2. The iris is immobile to light; but the similarity ends there. In glaucoma the iris, although immobile to light, is more *dilated* and the pupil *oval* in shape; the pattern of the iris is less affected and the iris seems to be pressed forward so as to be in contact with the posterior surface of the cornea—in other words, the anterior chamber is abolished.

If the attack of glaucoma is very acute the patient soon becomes collapsed and vomits frequently, almost simulating the condition of a person with a strangulated hernia.

Most important is the *tension* of the eyeball: in most cases of iritis it is unaltered or diminished, whereas in acute glaucoma it is *always* stoney hard. If, as is sometimes the case in acute iritis the tension is increased, the diagnosis must be made on other signs.

### To Nurses.

**We invite contributions from any of our readers, and shall be glad to pay for "Notes on News from the Nursing World," or for articles describing nursing experiences, or dealing with any nursing question from an original point of view. The minimum payment for contributions is 5s., but we welcome interesting contributions of a column, or a page, in length. It may be added that notices of appointments, entertainments, presentations, and deaths are not paid for, but that we are always glad to receive them. All rejected manuscripts are returned in due course, and all payments for manuscripts used are made as early as possible after the beginning of each quarter.**

### The King and Queen at Londonderry County Infirmary.

BY ONE OF THE NURSING STAFF.

We had been in a condition of suspense for the past three weeks expecting the promised visit, and wondering whether, although it was no part of the official programme, the King and Queen would inspect the wards. I need not say we had a busy time giving everything an extra shine, and this was the more difficult as the hospital is much broken up by alterations which are being made, and the dust and dirt consequent on its being full of workpeople. However, inside and out we made a brave show, and having put on our smartest uniform prepared to meet the King.

### The Queen and the Matron.

The matron and nurses were drawn up inside the front door, and, not being included in the official programme were naturally anxious as to whether they would get a glimpse of their Majesties. Their feelings may be imagined when, on the Queen alighting from the carriage, she at once passed through the reception committee, and having bowed to us, shook hands with the matron. The chairman and medical staff were then introduced, an address read and answered by the King, and his Majesty proceeded to lay a memorial stone.

### In the Children's Ward.

This over, he suggested a visit to the wards, which we had hardly ventured to hope for. The matron took their Majesties up in the lift and brought them to the children's ward, which was beautifully decorated by one of the benefactors of the hospital, who makes its adornment his special care. The King and Queen inquired about each case, and were especially interested in a little chap convalescent from tetanus. The little ones were quite at home with their visitors, and, indeed, so were all with whom they came in contact. Their Majesties also visited one of the women's wards and conversed with the patients.

### The King and the Pet of the Place.

The King espied a little lad of three, the pet of the place, sitting in a window sill, and, walking up to him, gravely saluted him. The child was shy and wrapped himself in the window curtain, which made his Majesty laugh heartily. The King and Queen then went over and inspected our extern, which is a beautifully fitted one. Afterwards they signed, with all their party, the visitors' book, and having shaken hands with the medical staff, went away.

### Deference to God's Sick Poor.

What struck us most of all was that from the moment they crossed the threshold of the hospital they seemed to lay aside all pomp and ceremony and to put themselves into the position of ordinary visitors; showing indeed to all an example of how a hospital should be visited by their beautiful courtesy and geniality. We found it difficult to realise that the kindly gentleman and sympathetic lady who had paid us such a visit were the King and Queen who had arrived at our door with such ceremony and who left us with equal ceremony, making us understand that the extraordinary affection in which they are held by their subjects is not a little due to (what we may call) the deference they pay to God's sick poor and those who attend upon them.

### The Nurses' Co-operation.

ITS LOSSES ON THE HOWARD DE WALDEN HOME.

In an article in The Hospital *Nursing Section*, February 28th, 1903, we pointed out that during the year 1902 there had again been a loss of upwards of £255 on the Nurses' Home and Club. A well-informed correspondent, who has an intimate knowledge of the facts, has supplied us with the following interesting particulars as to the present working of this Home. It will be remembered that about a year ago a new matron was appointed, who had had exceptional experience, and who is known in the nursing world as a lady who has been wonderfully successful in the economical management of the domestic expenditure of large institutions. When the new matron commenced her duties, our correspondent states, the home was in a very dirty condition, the books were badly kept, and the servants in a state of indiscipline. Further, the new matron was instructed that expense did not matter, providing efficiency was established throughout the home. From October 1901 to October 1902 the average number of nurses in the home appears to have been from 60 to 70 per week. At first the new matron did not increase the average number of residents, but during the last five months the number has increased, and it is stated that during May 1903 upwards of 290 nurses per week on an average used the home as a residence.

Our correspondent objects to a statement by the committee that the restaurant is conducted at a loss, because the figures for the month of May 1903 show a profit as follows:—Amount taken in the restaurant, £106; cost of food and all other stores, £85; profit for month, £21. The committee complained to the matron of the loss which they alleged was being made on the restaurant, and she replied that she could not make more profit, and suggested that they should get someone who understood restaurant management, and she would do without one of her assistants. This advice they consented to take, and appointed a manageress, at a larger salary than the matron was paid.

A short time ago the matron asked for a few extra beds to be put up in the Home, which it is stated could easily have been arranged; but the committee preferred to buy a flat in Langham Street, for which they are paying in rent about £150 a year. At the present time, and for some weeks past, this flat has been empty, though the rent is being paid in addition to the caretaker's salary. Our correspondent states that in these circumstances, instead of getting rid of the liability on the flat, the committee sent a letter last week informing the matron, just as she was starting on her holiday, that, "after careful examination of the financial position of the Howard de Walden Home, the committee have reluctantly come to the conclusion that a change in the management must be made," and that her services would not be required after October 1st. Had the committee sent in their resignations they would have shown commendable intelligence, but to change the matron just as she had made a profit on the restaurant is not business.

Our correspondent states that during the 10 months the present matron has had charge of the home she has, under the most trying circumstances, managed it exceedingly well, and improved it in every way, particularly in respect to cleanliness and order. How long will the nurses of this Corporation tolerate the abuses and losses resulting from the present dictatorship? It is for the nurses to determine whether they will act together and secure a capable and public-spirited committee to manage their business? Or whether they will permit things to drift on under incompetence and self-interest, until the Nurses' Co-operation fails, as it must then ultimately fail, by the withdrawal of public confidence and the boycott of the most capable and best nurses.

### Libel Action by a Hospital Nurse.

A SETTLEMENT BY AGREEMENT.

At the Birmingham Assizes on Friday, last week, before Mr. Justice Ridley and a special jury, an action was down for hearing in which Miss Alice L. Alcott, a nurse at the Hagley Green Hospital, Stourbridge, sought damages for libel from Miss Emma Loveys, matron of the Queen Victoria Nursing Institution, Bath Row, Wolverhampton.

Mr. A. T. Lawrence, K.C., and Mr. W. J. Disturnal (instructed by Messrs. Willcock and Taylor, Wolverhampton) were for the plaintiff, and Mr. H. H. Y. Stanger, K.C., and Mr. R. C. E. Plumptre (instructed by Mr. G. M. Martin, Wolverhampton) for the defendant.

Address of Counsel for Miss Loveys.

When the case was called on Mr. Stanger said: I am happy to say that your lordship and the jury will not be troubled with this case, the parties having come to terms. It is an action brought by the plaintiff in respect of two letters which were written by the defendant in reference to the character of the plaintiff and her qualifications as a nurse. My lord, the defendant is the matron of the Queen Victoria Nursing Institution in Wolverhampton, and she is a lady who is very highly esteemed by everyone who knows her. These letters were written in reference to applications as to the character and qualifications of the plaintiff. Your lordship will recollect, perhaps, having examined the pleadings, that what the defendant relied upon is privilege, and I think it is manifest that that is a defence which would be applicable to letters written in answer to applications as to the character of a person, and I am sure of this, my lord, that as long as it could be contended that Miss Loveys had written what she did from any malicious motive, or not believing that what she wrote was true, as long as that could be contended it would have been impossible to settle this action. It would have had to go to the jury, and I should have been no party, nor would Miss Loveys have been any party to any compromise which allowed it to be left for a moment in doubt as to whether she had acted properly or improperly. But your lordship will also remember that we have not justified, and therefore if the case had been before us the issue would have been not whether the statements in those letters were or were not true, but merely as to whether Miss Loveys had acted properly or improperly in making those statements. Now, my lord, inasmuch as I am able to say it will not be disputed—in fact I understand my learned friend (Mr. Lawrence) to make the acknowledgment—that Miss Loveys wrote from no improper motive whatever, there is really no difficulty about settling the matter. Now we have the fullest information and now that the matter has been entirely sifted I have had an opportunity of conferring with my learned friend, and I have endeavoured to confer with him and I am quite sure he has endeavoured to confer with me in a perfectly friendly and a perfectly reasonable spirit, because we were both anxious that while doing the utmost in the way of protecting our clients' interests we should adopt a course that was reasonable. I understand from my learned friend that he will state to your lordship that he has come here with a great number of testimonials and very strong evidence with regard to the character of the plaintiff. Now, my lord, in all the circumstances and with the full information that is now at our disposal, I am glad to say I have authority from Miss Loveys to entirely withdraw any imputations upon the plaintiff's character, either with regard to her character generally or with regard to her qualifications as a nurse, and Miss Loveys would be very sorry if these letters should be any bar in future to the success of the plaintiff in her professional career. The settlement that has been come to is satisfactory to the parties, I hope it will commend itself to your lordship, and I hope it will be thought the defendant has acted in this matter with every desire to do what was perfectly right from first to last.

Address of Counsel for Miss Alcott.

Mr. Lawrence, K.C.: I, with my friend, Mr. Disturnal, have considered the case on behalf of the plaintiff, who has adopted the profession of a nurse as a means of gaining her livelihood. The letters reflected upon her in her character as a nurse, and she brought this action to vindicate that character and to show that there is no ground whatever for any imputations upon it. My learned friend, with his accustomed good judgment, has made the matter clear, and has said that he withdraws all those imputations, and he withdraws them upon the fullest information which is now before him. My lord, nothing could be more satisfactory than that to my client, because my client is not desirous of imputing any malicious or any bad motives to the defendant. What she is desirous of showing is that her character is blameless, and that her qualifications as a nurse are beyond dispute. In these circumstances, my lord, I have the greatest pleasure in accepting the terms my learned friend has stated, and he has granted us such compensation for the wrong that has been done us that we feel we are amply justified in advising our client not to trouble your lordship and the jury with any further hearing of this case.

His Lordship: It is very satisfactory, I think.

Mr. Lawrence: Your lordship will grant us a judge's order if necessary?

Mr. Stanger: It will not be necessary, but of course we agree to that.

The record was then, by consent, withdrawn.

### Army Nurses at Dinner.

SPEECHES BY LADY ROBERTS.

A very interesting gathering took place on Wednesday, July 29th, at St. Andrew's House, Mortimer Street, when twenty-nine members of Queen Alexandra's Imperial Military Nursing Service, including the matron-in-chief, Miss Sidney Browne, and presided over by Countess Roberts, the Vice-President of her Majesty's Nursing Board, gave a farewell dinner to Miss Gray, the senior member of the late Army Nursing Service, who, on attaining the age limit of 55, has lately retired on her pension after 30 years' service. The guests of the evening were Miss Gray; her sister, Mrs. Eccles, who joined the service in 1873; Miss Monk, matron of King's College Hospital; and Miss Cave, matron of Westminster Hospital, who are members of Queen Alexandra's Nursing Board; Viscountess Downe, also a member of the board, was prevented by indisposition from being present.

Lady Roberts, in proposing Miss Gray's health, paid a high tribute to the splendid work she had done in her 30 years' service, and said that she carried with her the love and respect of all who had come in contact with her, and left behind her a bright example of devotion to duty and sympathetic care of "the soldiers of the King," which she (Lady Roberts) hoped the members of the new service would not be slow to follow.

In proposing the health of their Majesties the King and Queen, Lady Roberts pointed out the heavy debt of gratitude the nursing profession owes Queen Alexandra for the high estimation in which her Majesty holds nursing work, and all she has done to uphold the honour and dignity of the nursing profession. She said that nothing could tend to maintain a high tone in the profession so much as what the Queen is now doing by insisting that all who are admitted into Queen Alexandra's Imperial Military Nursing Service must be *sans reproche* as to character, and up to the highest level of training and efficiency.

It is hoped that this will be an annual dinner, and the first of many pleasant reunions of members of Queen Alexandra's Imperial Military Nursing Service and the Army Nursing Service.

### Miss J. A. Gray's Career.

Miss Gray, the guest of the evening, entered the service in 1873. She served in Zululand and was thanked by special command of the Queen for her services. From 1882 to 1883 she served in the Egyptian War, was commended for her services in Egypt, and decorated with the Royal Red Cross by the Queen. She was most conspicuous for unremitting attention to the sick during the Cholera Campaign in Egypt, received a letter conveying the Secretary of State's approval, and also a letter of thanks and appreciation from the Duke of Cambridge. She served in the Nile Expedition in 1885, Lord Wolseley reported most favourably upon her, and she was again recommended for the Royal Red Cross. For her services on the Gold Coast, during the war in 1896, she was again commended. Finally, Miss Gray served in the South African War from 1899 to 1902, was mentioned in despatches, and retired on her pension July 1903.

### Everybody's Opinion.

[Correspondence on all subjects is invited, but we cannot in any way be responsible for the opinions expressed by our correspondents. No communication can be entertained if the name and address of the correspondent are not given as a guarantee of good faith, but not necessarily for publication. All correspondents should write on one side of the paper only.]

### THE ONE-YEAR NURSE.

"An Infirmary Nurse" writes: Will you, though rather late in the day, kindly allow me to say a few words on the subject of the one-year trained nurses? In the first place, women who offer themselves for one year's training are generally thoroughly in earnest, and their ages vary from 25 to 35, the most sensible time in a woman's life. When sent to training schools such as West Derby Union Infirmary and Brownlow Hill, Liverpool, attending lectures and operations, working under the supervision of capable medical men, hearing lectures from matrons, and attending classes in practical work, such as bandaging, application of splints, testing urine, and knowing that they have only one year to learn practical and theoretical nursing, they will give their whole interest to the work, and will not allow one day to pass without learning something new. I maintain that a one-year nurse is not badly equipped for the work when she leaves at the end of her training, providing she is fairly capable and intelligent. It is not to her advantage to train for one year only, for she cannot hope to become matron, superintendent, or sister in a large hospital, or take a large salary. So her motive must be to nurse the sick poor only. I do not agree with Mrs. Richmond when she proposes that on a medical man should devolve the responsibility of saying whether a patient requires the skill of a one-year's nurse or three-years' nurse. He should rather use his own judgment in judging if the nurse was capable in carrying out his instructions faithfully and intelligently, and in trusting her to give him a true account of observations during the time she was on duty. This is, I believe, the duty of a nurse; and this a nurse of one year's training in a large infirmary is quite capable of doing. She is also capable of taking notes of case and giving correct statement of facts. One of the first lectures a matron gives to her probationers is on observations. It is not merely being so many years in a hospital which makes a good nurse. Character and capabilities should also be taken into consideration.

"A Workhouse Infirmary Nurse" writes: The statement made by the matron of Luton Workhouse that in a certain district out of 40 odd workhouse matrons more than half were trained nurses, must have surprised many superintendent nurses with workhouse nursing experience and have led them to wonder how many of these workhouse matrons could produce evidence of satisfactory training in reliable training schools. If so, why are they raising such an outcry and holding meetings all over the country to protest against the report of the Departmental Committee *re* superintendent nurses when that report distinctly states that, with the necessary qualifications and midwifery training a workhouse matron may also hold the post of superintendent nurse. I am reminded of an incident which occurred a short time ago. When staying for a few days with a workhouse matron, a friend of mine, we called one afternoon on the matron of an adjoining workhouse, and during conversation this matron informed me that she was a trained nurse. On being taken round the wards I was surprised at many things I saw though I said nothing till later on. When speaking of the matter to my friend and asking her if she knew where this matron was trained, I learnt that she had been an assistant in a country union where only two nurses were kept and had married the porter there. I gathered that she had never even worked under a trained nurse. If workhouse infirmaries are to be improved, it is to be hoped that the Local Government Board, before sanctioning the appointment of the workhouse matron as superintendent nurse also, will insist on her possessing the full qualifications necessary for such a post. It is well known among experienced nurses that workhouse masters and matrons are generally the advisers of the Guardians (unfortunately, in many cases), and in making an appointment some Boards do not even trouble to consult the doctor as to a nurse's capabilities. A workhouse matron might easily persuade such a board to give her the management of the infirmary and nurses if her knowledge of nursing is ever so slight or rusty. Personally, and speaking from practical experience, I do not believe in the joint appointment of workhouse matron and nurse, except for the very small workhouses where the wards are in the workhouse; in an infirmary of, say, from 50 to 100 beds, both patients and nurses must be all the better for the supervision of a trained nurse who is on the spot. A workhouse matron usually has a family, and in many cases her house is quite a little distance from the infirmary. This means that she naturally spends a good deal of her time at her house—for instance, meal times, her evenings, and of course she would never be in the infirmary at night unless summoned. Most nurses will, I think, agree with me that this cannot be good for the infirmary or for the (more often than not) untrained and inexperienced nurses working in them.

### PRIVATE NURSES AND WINE.

"In Medio Tutissimus Ibis" writes: With "District Nurse" I would like to ask why it is that a woman who can be trusted with the care and responsibility of a sick person, cannot also be trusted to take a glass of wine—or rather, cannot be trusted to limit herself to a proper number of glasses?—for that is, after all, what all this "no wine" rule comes to. I am a "total abstainer," and was one long before I joined the nursing profession, though not because I fear to trust myself near a bottle. But I know many nurses who are better able to bear the fatigue of nursing if they have a glass of wine at lunch and at dinner, and who miss it when they cannot have it. And I think that the rules of a nursing institute forbidding the nurses any stimulant are insulting to the women for whom they have been framed. On principle, I would refuse to sign such an agreement. I was once asked by the grandmother of a little patient, when she was leaving me for the first night, whether I would like a little wine left for my own use. I thanked her, but declined, adding that I was an abstainer. Whereupon the old lady flung her arms round my neck and assured me, with more vehemence than tact, that she could "now rest easy in her mind," and that "now" she would be "perfectly sure that little Bobby would want for nothing all night." The dear old lady—and I count her among my dearest friends now—was quite unaware that her remarks had not been exactly tactful, nor could they by any stretch of imagination be considered flattering to my vanity, seeing that, while excepting me, on my own word, she undoubtedly still looked upon the remaining members of my profession as worthy followers of the immortal Sairey Gamp. As soon as I recovered from the shock of the embrace, I felt first amused, then angry, then puzzled. And puzzled on the subject I have been ever since —puzzled to know why nurses—the women of all others, whose lives have come for a certain period under steady discipline, and whose daily work is in itself elevating and ennobling—should be the class of women who cannot be trusted to take their wine in moderation. The only solution I can find for the puzzle is, that in spite of the lapse of years since Sairey Gamp was abroad the shadow of her portly form still rests on the nursing profession. I hope that the day is not far distant when it will be lifted.

### Appointments.

[No charge is made for announcements under this head, and we are always glad to receive, and publish, appointments. The information to insure accuracy should be sent from the nurses themselves, and we cannot undertake to correct official announcements which may happen to be inaccurate. It is essential that in all cases the school of training should be given.]

Birmingham General Hospital.—Miss Emily Garside has been appointed home sister and night sister. She was trained at Liverpool Royal Infirmary, and has since been sister at Salop Infirmary, Shrewsbury, temporary matron at Newport Home and Hospital, and night superintendent at the Children's Infirmary, Liverpool.

Bolton Infirmary and Dispensary.—Miss Rundle has been appointed sister. She was trained at the Royal Hospital, Portsmouth, and has since been nurse in a genealogical home in London, and sister at the Stockport Infirmary.

Chester Isolation Hospital.—Miss Madeline McArdle has been appointed matron. She was trained at the Birkenhead Infirmary, and has since been charge nurse at the Park Hospital, Lewisham, London; night superintendent at City Hospital, Liverpool; and deputy matron at City Hospital, Fazakerley.

Clara Baroness de Hirsch Convalescent Home, Hampstead Heath.—Miss Rose L. Shappere has been appointed matron. She was trained at Prince Alfred Hospital, Melbourne, and has since been sister at the Perth Hospital, Western Australia, and night sister at the Adelaide Hospital. She has also done private nursing for many years in Australia, and has been acting superintendent sister on transport and hospital ships during the South African war.

Craigleith Hospital, Edinburgh.—Miss Agnes Lang has been appointed charge nurse. She was trained at Bolton Union Infirmary, and has also done private nursing in Great Grimsby.

Cumberland Infirmary, Carlisle.—Miss E. M'Cummins has been appointed matron. She was trained at the London Hospital, Whitechapel, where she was afterwards staff nurse for two years. She has since been ward sister and assistant matron at the Royal Infirmary, Bristol. During this period she was lecturer on nursing for the Merchant Venturers' Technical College, Bristol, during one session.

Farmfield Reformatory, Charlwood, Horley.—Miss E. Forsyth has been appointed lady superintendent. She was trained at the Royal Hospital for Sick Children, Edinburgh, and at the Royal Infirmary, Dundee. She has since been sister at the Mildmay Hospital, Bethnal Green, night superintendent at the Southwark Infirmary, home sister at the London Temperance Hospital, and matron of St. Anne's Home, Herne Bay.

Infectious Diseases Hospital, Blackpool.—Miss Sarah Collie has been appointed night charge nurse. She was trained at Greenock Infirmary.

National Hospital for Consumption, Newcastle, County Wicklow.—Miss Gertrude Heald has been appointed sister, and Miss Margaret Smith staff nurse. Miss Heald was trained at Bradford Infirmary, and has since been sister at St. Mary's (Islington) Infirmary, and Woolwich Infirmary. Miss Smith was trained at Galashiels Cottage Hospital, and the National Hospital for Consumption for Ireland. She has since been assistant nurse at Dover Fever Hospital, nurse at the Small-pox Hospital, Dartford, and staff nurse at the Liverpool Sanatorium, Frodsham.

Pontypridd Workhouse Infirmary.—Miss Bessie Mackay has been appointed charge nurse. She was trained at North Devon Infirmary, Barnstaple, and has since been charge nurse at Halifax Union, on the private nursing staff of the Royal Devon and Exeter Hospital, and nurse at Liverpool City Fever Hospital.

Rotunda Hospital, Dublin.—Miss Margaret P. Love has been appointed night superintendent. She was trained at the Western Infirmary, Glasgow, and at the Rotunda Hospital, Dublin.

Royal Cornwall Infirmary, Truro.—Miss Esther J. Pearce has been appointed sister. She was trained at Westminster Hospital, and has since been Queen's nurse for two years, charge nurse at Ilkeston Accident Hospital, and charge nurse at Saffron Walden Hospital. She holds the L.O.S. certificate.

### Presentations.

Crumpsall Workhouse Infirmary.—Last Friday, on the occasion of her resignation, after seven years' residence, Miss Entwistle was presented by the nursing staff of the Workhouse Infirmary, Crumpsall, with a handsome silver set of toilet requisites. Miss Entwistle was trained at Crumpsall, where she was afterwards in charge of wards. For nearly three years she has been house sister. The maids of the institution also presented her with a handsome Queen Anne silver sugar basket.

Manchester Hospital for Consumption.—An interesting little event at the Manchester Consumption Hospital the other day was the presentation to Miss H. M. Morris, the senior charge nurse, who is leaving to take up a superior post, of a gold watch by the honorary staff in recognition of her services to the institution, a gold curb bracelet from the in-patients, and a gold chain and pendant from the former patients, in token of esteem and regard for her uniform kindness to them.

### TRAVEL NOTES AND QUERIES.

Accommodation in Chamouix (A. L.).—First, as to hotels; you give me no idea of what you wish to pay, but Grand Hôt Couttel will take you from 8 francs per day, so will Hôt des Alpes; Hôt Suisse from 7 francs, Hôt de la Paix and Hôt Croix Blanche from 5 francs. There is a great deal to see at Chamouix, and I should advise your staying there the whole week rather than cutting up your time by a fresh move. One whole day will be occupied in seeing the Mer de Glace and the Flégère. Another day for the Glacier des Bossons. Then there is the Aiguille de la Tour, Pavillon de la Pierre Pointue, and the Grandes Mulets. That is probably as high as you will wish to go unless you are good mountaineers. If you decide to divide your time I think it would be well to go to Martigny Hôt Aigle, 6 francs, or Hôt Grand St. Bernard, from 5 francs, for the sake of the diligence journey, 8½ hours, over the Tête Noir. You also visit the Hospice of St. Bernard from Martigny. In the season the Hospice is often very full.

### Echoes from the Outside World.

### The King and the Irish People.

On Monday copies of the address from the King to the Irish people were posted throughout the sister island. His Majesty says that he desires on leaving Ireland to express to his Irish people how deeply he has been touched by the kindness and goodwill which they had shown to the Queen and himself. For a country so attractive and a people so gifted, they cherish the warmest regard, and it is therefore with supreme satisfaction that his Majesty has during the visit so often heard the hope expressed that a brighter day is dawning upon Ireland. He will eagerly await the fulfilment of this hope, and adds that its realisation will largely depend upon the development of self-reliance and co-operation, upon better and more practical education, upon the growth of industrial and commercial enterprise, and upon the increase of mutual respect and toleration.

### Incidents of the Royal Visit.

Last week the King and Queen, accompanied by Lord and Lady Dudley, landed at Bundorragha, and started off in motor cars to inspect the district, leaving Princess Victoria and the Countess of Gosford to fish for salmon. At Glenagilma and Bay View their Majesties inspected some looms set up in cottages, and the Queen bought 35 yards of Connemara tweed which two boys were weaving, the King expressing his satisfaction at her purchase. The Queen also went into a typical Connemara cottage—a building only a few feet square—showing a kindly interest in the bare-legged children who happened to be at tea. Then through mud and slush the royal party tramped to a wretched thatch-roofed cabin where an old man of 80, partly bedridden, had lived for more than half a century. As the cabin was some way from the road, the King and Queen walked through the soft boreen, their passage being challenged by the pig. Mrs. Keerigan received them, but had no idea who they were. Whilst the Queen went in to talk to old Patrick about his ailments, the King chatted with his wife. Afterwards when she found out the identity of her visitor, "Shure," she said, "the King was much cheerier and nicer than many a man who has got a baste to drive on the road before him." At the next cottage the woman had to drive away the chickens before she wiped the chair for the Queen to sit upon.

On Thursday the King received an address, and then started out for a motor ride to Recess accompanied by a unique escort of Connemara farmers on their shaggy ponies, 100 strong. They visited the famous marble quarries, and here the Queen noticed an ill-clad, emaciated-looking, peasant woman, and calling Viscount Churchill gave him half a sovereign to hand to the woman. The poor soul looked first extremely astonished and then burst into tears. At the station a peasant woman approached and handed the Queen a petition asking for a remission of the unexpired portion of six months' imprisonment her husband was undergoing. The Queen made inquiries as to his previous character, and then told her that the King would grant her request and order his release.

On Friday the Royal party landed at Galway and went by train to Kenmare, whence they drove to Dereen on a visit to Lord and Lady Lansdowne. The Galway Salmon Fishery sent a salmon of 20 lb. weight, just freshly caught, for the King's acceptance. On Saturday morning the Royal yacht arrived at Queenstown, and then his Majesty's ship *Vivid* proceeded nine miles up the river to Cork. Here the King presented new colours to two Irish regiments, addressed the troops, and drove on to the Cork Exhibition, where several addresses were presented. After being entertained to luncheon in the Exhibition they inspected the exhibits, and later embarked again on board the yacht for Cowes, where they arrived on Sunday evening. On Monday the King unexpectedly landed at Cowes, and on Tuesday he opened the Royal Naval College at Osborne. In the afternoon the Royal party went for a cruise up and down the Solent on the *Britannia*. Before quitting Queenstown the King conferred a Knight Commandership of the Victorian Order on Mr. Horace Plunkett, and the Lord-Lieutenant of Ireland received a sum of £500 from the Queen which she wished distributed "amongst the poorest in Dublin and other parts of the country where most needed."

### The Election of the New Pope.

Sixty-two Cardinals arrived in carriages at the Vatican on Friday to attend a solemn service prior to entering the Conclave, where they are incarcerated till they have elected the new Pope, the Cardinal who receives two-thirds of the total votes. Afterwards they made their way to the Sistine Chapel, each wearing a mantle of purple material, which is never worn on any other occasion of their lives. They sat in stalls under a canopy, emblematical of temporal power. During the interregnum between the death of one Pope and the election of another, every Cardinal is joint sharer in the administration, and honours due to the Pontiff alone are paid to each, but the moment one of their number is selected all the canopies collapse with a flapping sound, except that of the new Pope, and all power again becomes vested in one man alone. Here the 62 Cardinals took an oath to observe the regulations laid down by Gregory XV., and later on all attendants and servants swore on the Gospel and the Crucifix never to reveal anything they might learn in the Conclave. Afterwards the Cardinals took possession of their cells. In all 365 persons were shut in, including four masters of the ceremonies, three doctors, several druggists, four barbers, one carpenter, one locksmith, several tailors, besides 11 cooks and 24 valets. On Saturday morning smoke issued from the chimney connected with the stove where the voting papers are burnt after perusal. As the smoke was thick and heavy, the paper being purposely mixed with damp straw, the thousands assembled outside the Vatican knew that the first voting had been unsuccessful. Had a decision been arrived at the paper would have been burnt without the straw, and so given forth only a fine thin smoke. In the afternoon about 6 o'clock smoke again emerged from the slender iron pipe, and twice the same occurred on Sunday and Monday. On Tuesday morning Cardinal Sarto was chosen, and a few minutes before 12 the new Pope appeared on the inner balcony of St. Peter to give his benediction to the people. His Holiness was received with acclamation by an enormous crowd. At one time as many as 50,000 people, including gaily-dressed ladies in carriages, had assembled on the Piazza San Pietro.

### Pope Pius X.

Cardinal Guiseppe Sarto, who thus succeeds to the throne of St. Peter, as Pius X., is 68 years old, and was born at Riese, in Northern Italy, on June 2, 1835. He was ordained in September, 1858, and until 1875 acted as parish priest in various parishes of the Venetian diocese. He is known as an earnest, talented, and hard-working ecclesiastic, but his elevation to the Papacy was not considered within the range of possibility. On being appointed in 1884 Bishop of Mantua, he devoted himself to the task of improving the tone of the diocesan clergy, and by his tact and genius for organisation he effected notable changes. He is simple in his personal habits, modest and gentle in his bearing towards others, and has never entered the stormy arena of political controversy. In 1893 he was created Cardinal Patriarch of Venice.

### For Reading to the Sick.

"HOLD THOU MY HAND."

Hold my hand, and I can bear it,
O Thou Fellow-Sufferer;
There is no pain, if Thou share it,
Quite impossible to bear.

Hold my hand, and let no other
Comfort ever intervene;
Thou art closer than a brother,
Let no brother come between.

Hold my hand, that pressure feeling,
I shall bravely bear it all;
Though my senses may be reeling,
I might faint, but never fall.

Hold my hand, when all is over
I shall ope my tired eyes,
And my perfect health recover
At Thy side in Paradise.

*Anon.*

Oh, look not at thy pain or sorrow, how great soever; but look from them, look off them, look beyond them, to the Deliverer! whose power is over them, and whose loving, wise, and tender spirit is able to do thee good by them. The Lord lead thee, day by day, in the right way, and keep thy mind stayed upon Him, in whatever befalls thee; that the belief of His love and hope in His mercy, when thou art at the lowest ebb, may keep up thy head above the billows.

Will we take Him at His word? . . . Will we learn the lesson which comes so slowly, that in Him and in the consciousness of His love there is rest here and now, rest in the burden and heat of the day, rest in the long afternoon, rest in the dreary routine, rest in the tiresome details that tax our attention and weary our brains, rest in the exacting tasks; amid the stones and the rough places, the disappointments and the trials, the weariness and the loneliness and the uncongenial companionship, there is still rest in Christ, rest at His feet in the attitude of obedience, rest in His heart in the felt assurance of His love and interest, rest in deliverance by His life from the real torment, the torment of self, the agony of self-consciousness, from which no one and nothing else but Christ Himself, and a maintained union with Christ, can ever deliver us.

Robert Eyton, *The True Life.*

To die and be at rest
Beyond the power of sin,
Love and abiding guest
The ransomed Soul within.

To die and be at rest—
For this our natures crave,
The last home of the Blest,
The World beyond the grave.

To die and be at rest—
It is a Christian prayer,
For Death is God's Behest,
Christ and His Saints are there.

*H. Oxenham.*

### Notes and Queries.

**FOR REGULATIONS SEE PAGE 219.**

*Dispenser.*

(189) Can you tell me of any Hospital or Mission in London where I could gain employment as a dispenser? Am fully qualified, and would like to sleep at home.—*H. M. M.*

We are sorry not to be able to help you. Advertising and answering advertisements is, we fear, your only course.

*Red Cross.*

(190) Will you kindly tell me why the Red Cross is so generally used as a symbol of everything connected with nursing?—*A Nurse.*

Because the Red Cross was the symbol and badge arranged by the Treaty of the Convention of Geneva, February 9th, 1863, to ensure those wearing it protection whilst engaged in duties connected with the wounded in battle.

*District Nurse.*

(191) The town in which I live is desirous of adding a district nurse to the present staff. Can you kindly tell me on what lines country district nursing is worked, or give me the name of a society in good working order from which I could make inquiries?—*Sister Francisca.*

Write to the Superintendent-General, the Queen Victoria's Jubilee Institute for Nurses, Queen Katharine's Precincts, Regent's Park, N.W.

*Massage.*

(192) Will you kindly tell me if there is any centre, excepting London, in any part of the United Kingdom where I could attend classes for massage and prepare for examination? And if they would be as good as London?—*J.*

The Secretary of the Incorporated Society of Trained Masseuses, 12 Buckingham Street, Strand, W.C., would doubtless be able to help you.

*Army Nursing.*

(193) Will you kindly tell me the best general hospital at which to train for Queen Alexandra's Imperial Nursing Service?—*W. E.*

The certificate of any of the more important schools which give a three years' training would be recognised as qualifying for admission.

*Colosseum Association.*

(194) Will you kindly give me the address of the Nurses' Colosseum Association, London.—*Nurse H.*

The address is 7 Colosseum Terrace, Regent's Park, N.W.

*Clerkship.*

(195) Will you kindly tell me how I could gain information regarding clerkships in London hospitals? I should very much like to obtain a situation as such, but I do not know to whom to apply.—*J. B.*

Advertise in journals connected with hospital administration.

*Maternity Nursing.*

(196) I have just finished my two months' course of training at the City of London Lying-in Hospital, and I am now open to an engagement. Will you kindly tell me the best means of obtaining employment?—*K. R.*

The only methods of obtaining employment are either by private introductions or by advertisement.

*Home.*

(197) Will you kindly tell me where a child subject to fits and at times quite imbecile, could be received for a small weekly payment?—*Leamington.*

Write to the National Association for Promoting the Welfare of the Feeble-Minded, 53 Victoria Street, S.W.

*Hospital Training.*

(198) I am anxious to be trained as a hospital nurse. I am not yet 19, am I too young?—*Helen W. and R. K.*

You must wait until you are 23 for general training.

**Standard Nursing Manuals.**

"The Nursing Profession: How and Where to Train." 2s. net; 2s. 4d. post free.

"Nursing: Its Theory and Practice." (Revised Edition). 3s. 6d. post free.

"Surgical Ward Work and Nursing." (Revised Edition). 3s. 6d. net.; 3s. 10d. post free.

"Practical Handbook of Midwifery." (New Edition). 6s. net; 6s. 3d. post free.

"Notes on Pharmacy and Dispensing for Nurses." 1s. post free.

"Fevers and Infectious Diseases." 1s. post free.

"The Art of Massage." (New Edition). 6s. post free.

